# Supplementary material for: Aza-Reversine Promotes Reprogramming of Lung (MRC-5) and Differentiation of Mesenchymal Cells into Osteoblasts
Source: Materials (Basel). 2021 Sep 17;14(18):5385. doi: 10.3390/ma14185385 (PMC8467999; doi:10.3390/ma14185385)
Supplement: Supplementary file 1 [file materials-14-05385-s001.zip › materials-1338530-supplementary.pdf]

Supplementary Materials

Aza-Reversine Promotes Reprogramming of Lung (MRC-5) and Differentiation of Mesenchymal Cells into Osteoblasts

Fani Tsitouroudi [1], Vasiliki Sarli [1,*], Dimitrios Poulcharidis [1], Maria Pitou [1], Alexandros Katranidis [2] and Theodora Choli-Papadopoulou [1,*]

[1] Department of Chemistry, Aristotle University of Thessaloniki, University Campus, 54124 Thessaloniki, Greece
[2] Institute of Biological Information Processing IBI-6, Forschungszentrum Jülich (FZJ), 52425 Jülich, Germany
* Author to whom correspondence should be addressed.

Correspondence: tcholi@chem.auth.gr (T.C.-P.); sarli@chem.auth.gr (V.S.)

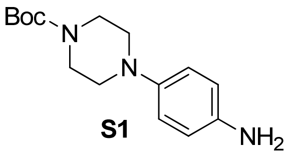

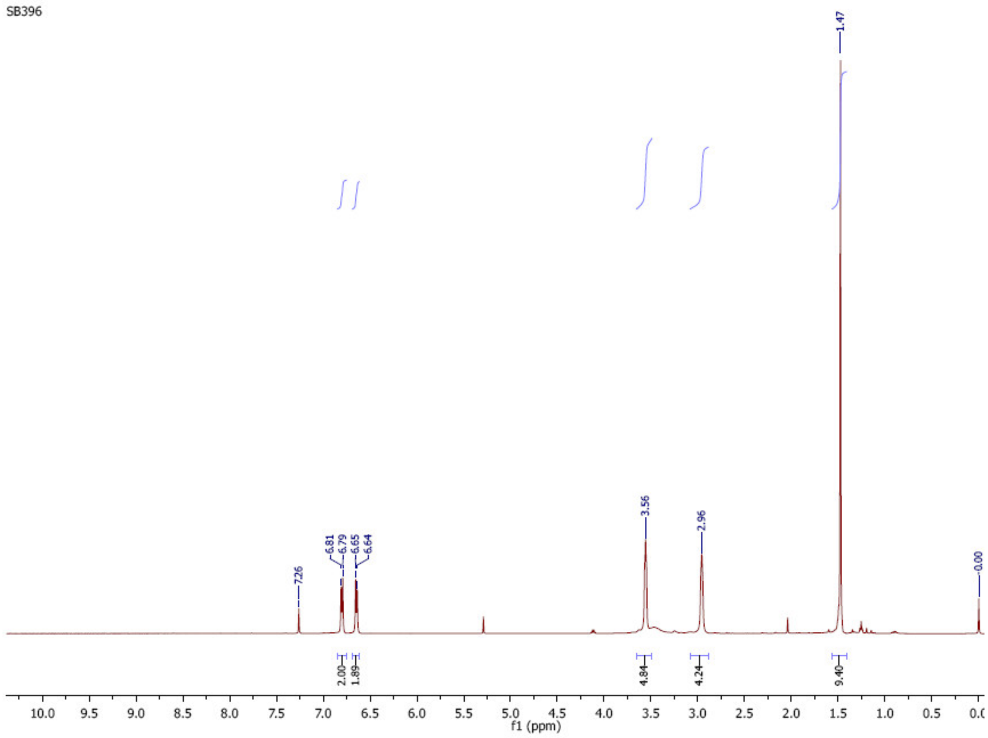

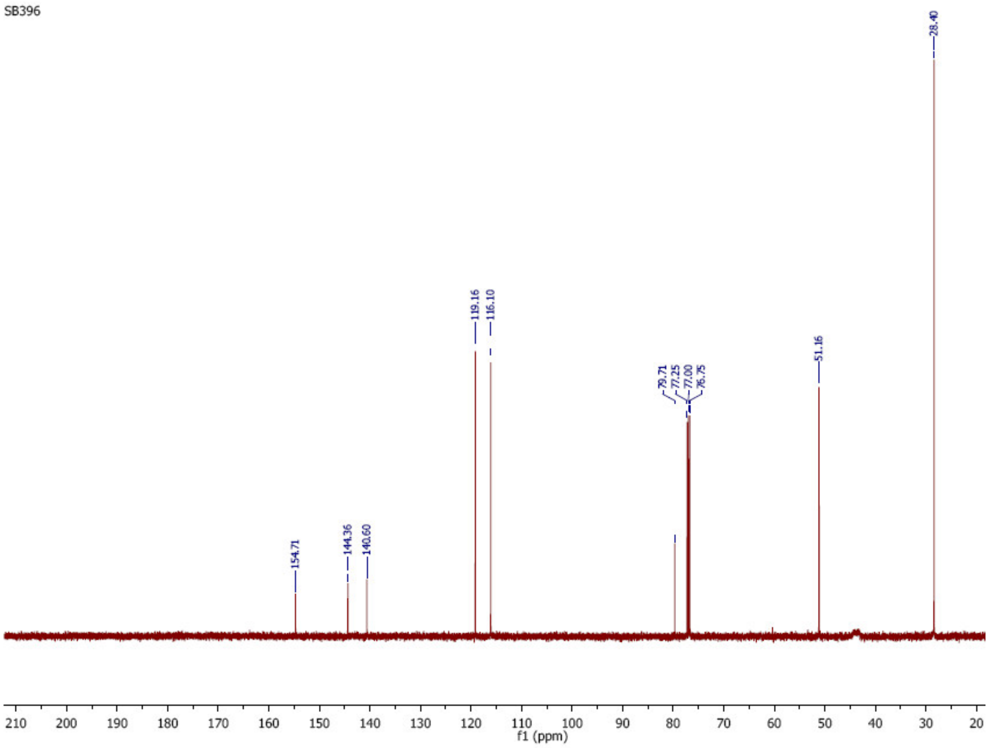

Figure S1. ^{1}H and ^{13}C NMR spectra of S1.

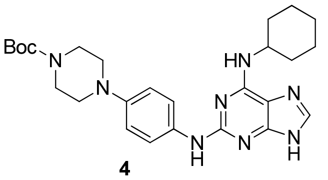

4

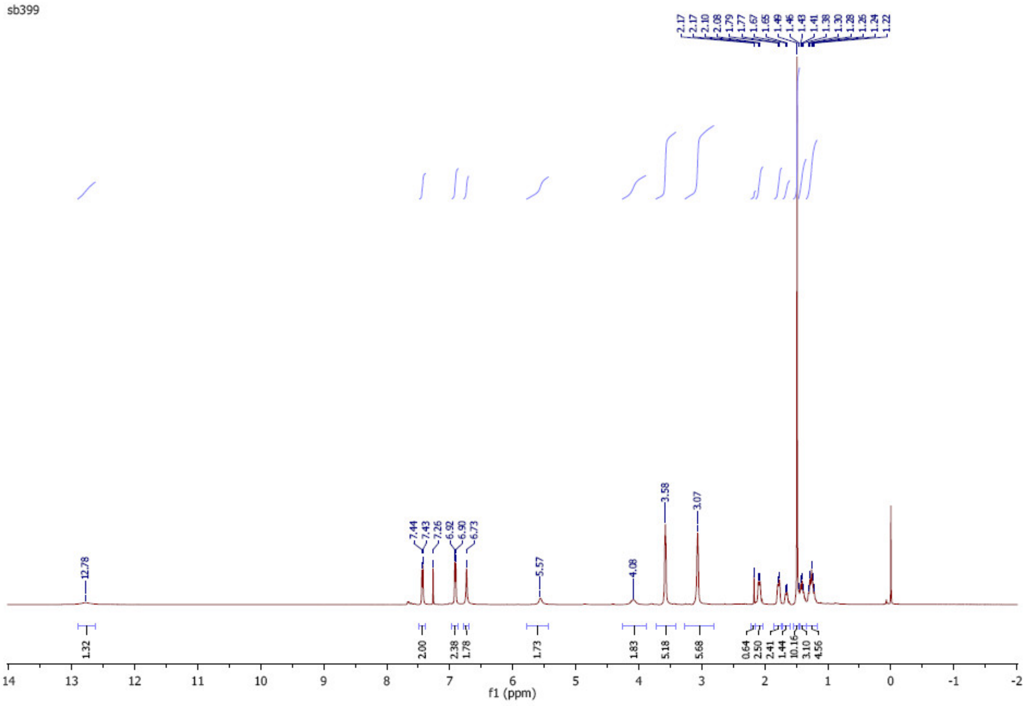

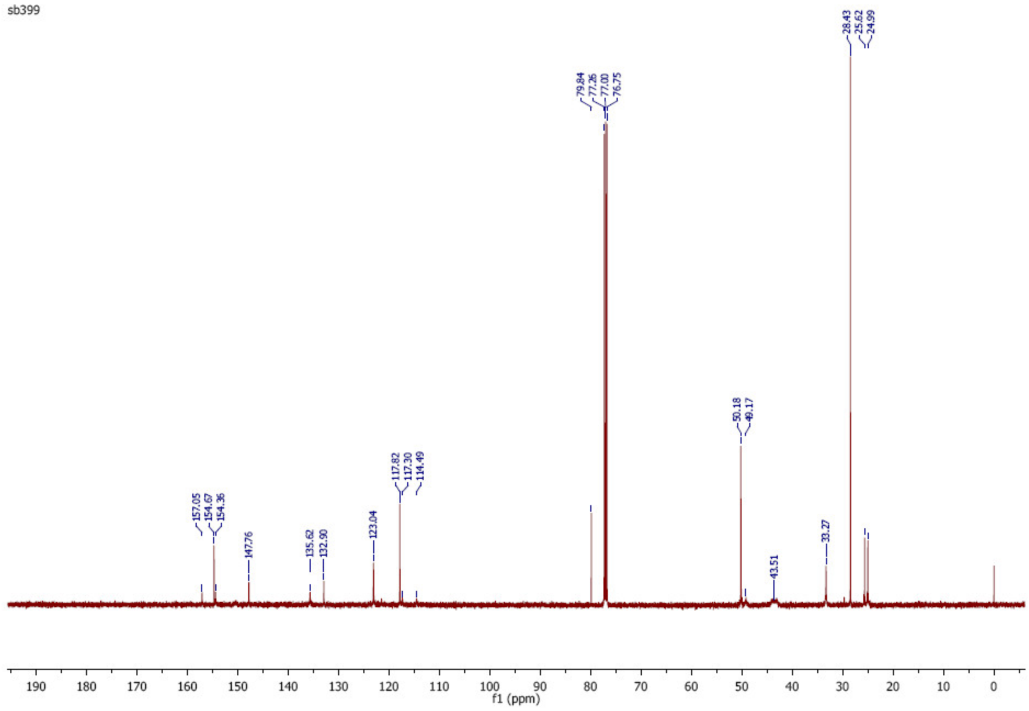

Figure S2. ^{1}H and ^{13}C NMR spectra of 4.

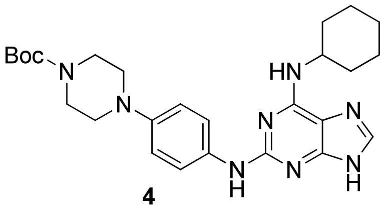

4

gDQCOSY

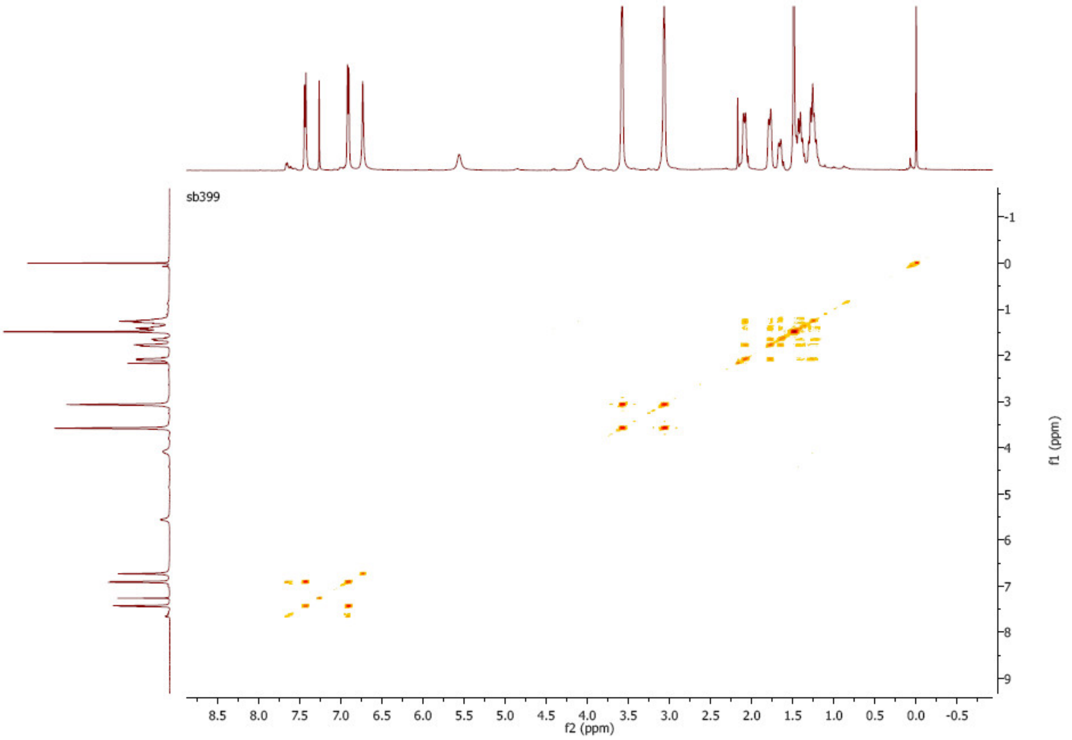

Figure S3. gDQCOSY spectrum of 4.

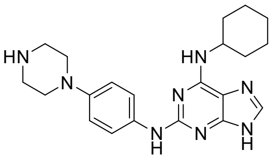

2

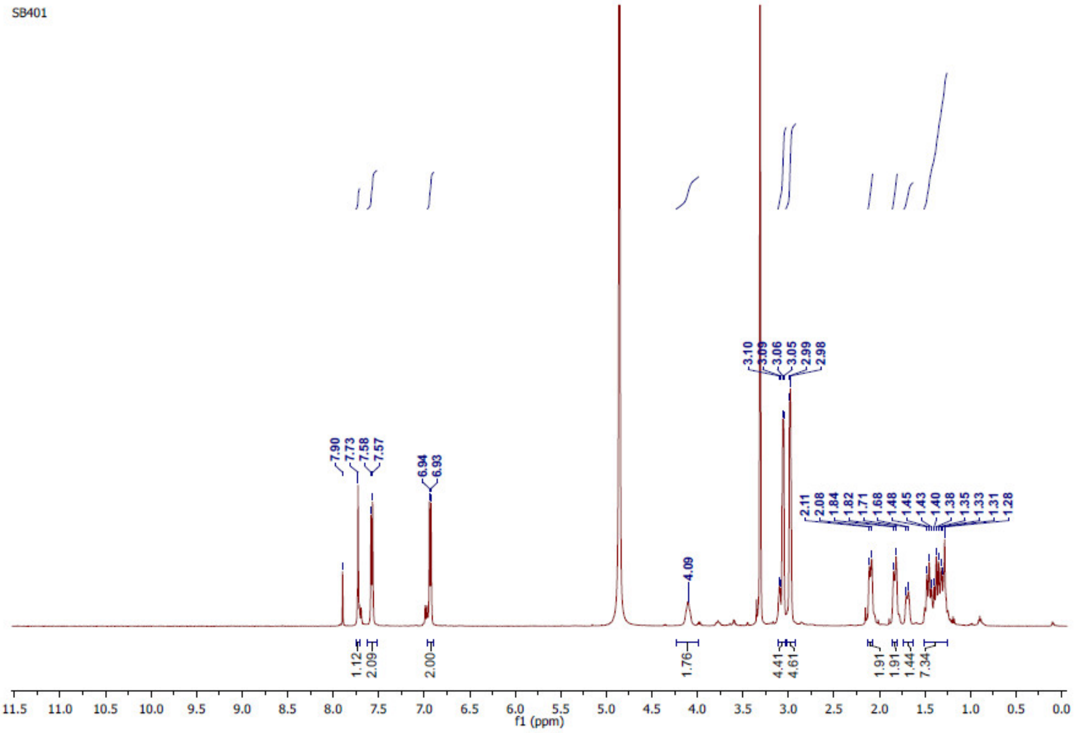

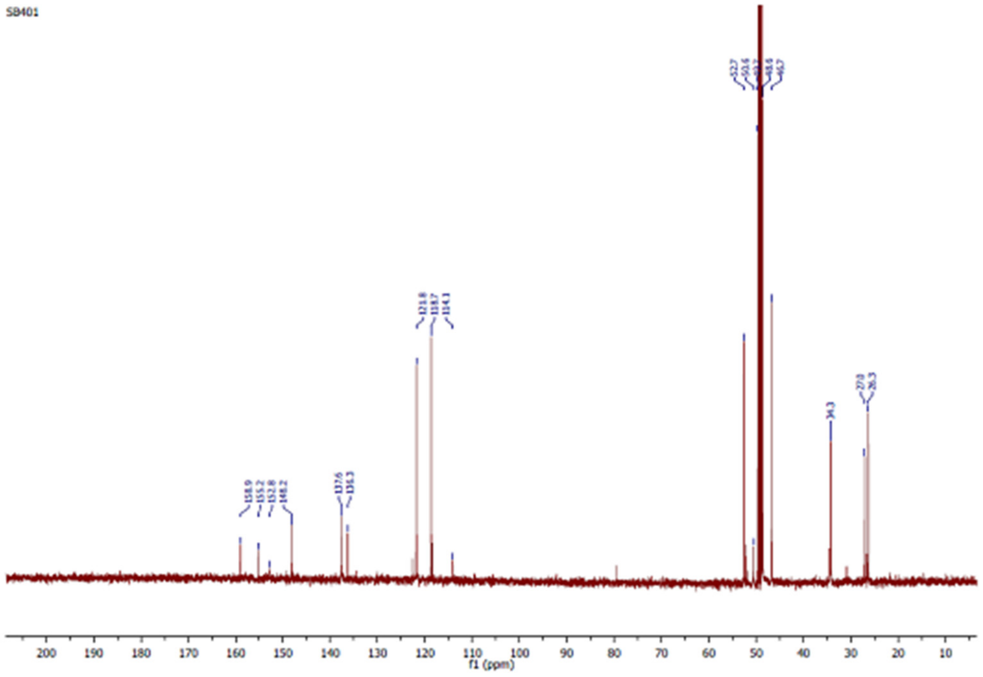

Figure S4. ^{1}H and ^{13}C NMR spectra of 2.

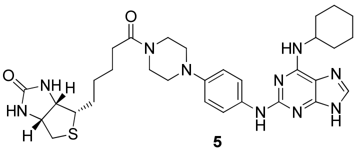

5

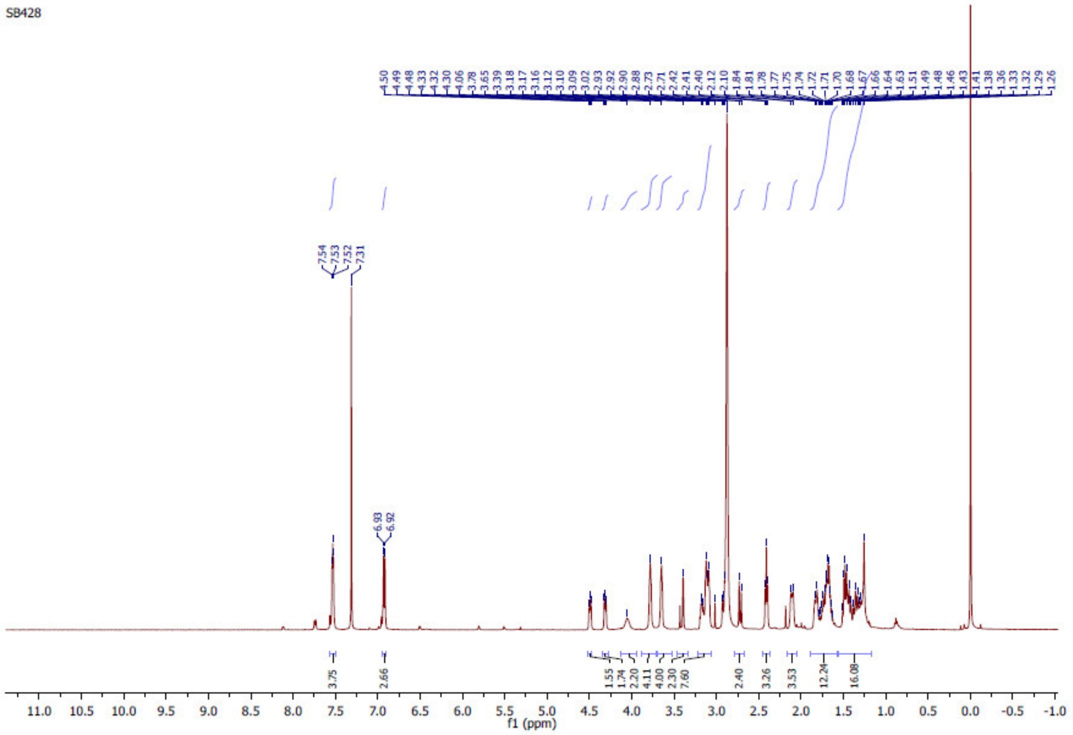

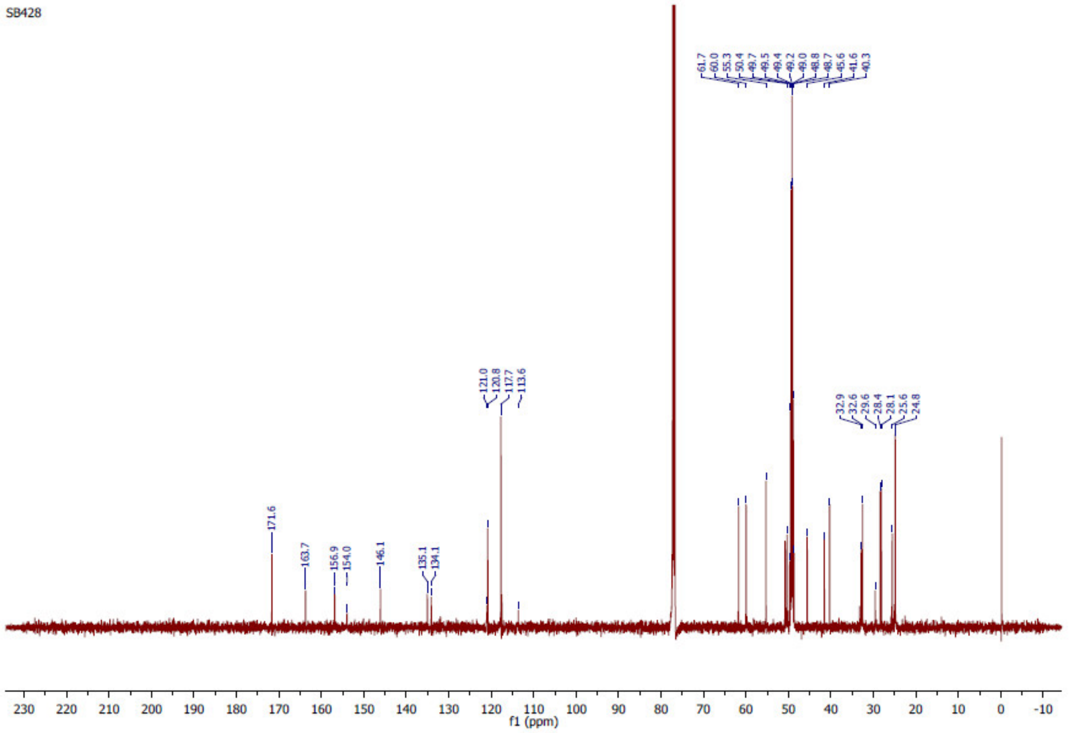

Figure S5. ^{1}H and ^{13}C NMR spectra of 5.

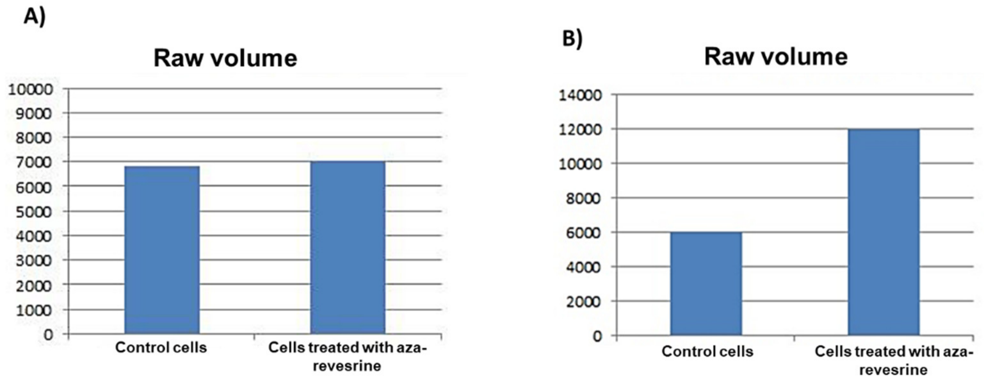

Figure S6. Quantification of band intensity after rt-PCR (a) Gus-B, (b) osteocalcin.
